# An assessment of zinc oxide nanosheets as a selective adsorbent for cadmium

**DOI:** 10.1186/1556-276X-8-377

**Published:** 2013-09-05

**Authors:** Sher Bahadar Khan, Mohammed M Rahman, Hadi M Marwani, Abdullah M Asiri, Khalid A Alamry

**Affiliations:** 1 Center of Excellence for Advanced Materials Research (CEAMR), King Abdulaziz University, P. O. Box 80203, Jeddah 21589, Saudi Arabia; 2 Chemistry Department, Faculty of Science, King Abdulaziz University, P. O. Box 80203, Jeddah 21589, Saudi Arabia

**Keywords:** Zinc oxide, Nanosheets, Metal uptake, Cd(II), Environmental applications

## Abstract

Zinc oxide nanosheet is assessed as a selective adsorbent for the detection and adsorption of cadmium using simple eco-friendly extraction method. Pure zinc oxide nanosheet powders were characterized using field emission scanning electron microscopy, energy dispersive spectroscopy, X-ray diffraction, X-ray photoelectron spectroscopy, and Fourier transform infrared spectroscopy. The zinc oxide nanosheets were applied to different metal ions, including Cd(II), Cu(II), Hg(II), La(III), Mn(II), Pb(II), Pd(II), and Y(III). Zinc oxide nanosheets were found to be selective for cadmium among these metal ions when determined by inductively coupled plasma-optical emission spectrometry. Moreover, adsorption isotherm data provided that the adsorption process was mainly monolayer on zinc oxide nanosheets.

## Background

With the development of industry and economy, environmental problem becomes more and more serious day by day [1-3]. Due to certain man-made activities, numerous hazardous compounds and heavy metals are introduced into the environment which is a concerning matter for monitoring agencies and regulation authorities [4-6]. Among these pollutants, toxic metals are the most sever pollutants and main environmental threat which instigate too many serious public health and cost-cutting problems [7,8]. Cadmium is known to be as highly toxic as probably carcinogenic for humans and is listed as the sixth most poisonous substance jeopardizing human health. Cadmium is introduced into water bodies from different sources, for example, smelting, metal plating, cadmium-nickel batteries, phosphate fertilizers, mining, pigments, stabilizers, alloy industries, and sewage sludge. The harmful effects of Cd(II) involve a number of acute and chronic disorders such as gastrointestinal irritation, vomiting, abdominal pain, diarrhea, renal damage, emphysema, hypertension, and testicular atrophy [9,10]. Therefore, separation and determination of Cd(III) in different matrices have continued to be of import.

In addition, the development of simple, rapid, and efficient methods has become of interest for monitoring metal ions in the environment. Several analytical methods have been applied to analyze metal ions in aqueous solutions [7,8]. However, analytical methods cannot directly measure metal ions, in particular at ultra-trace concentration, in aqueous systems due to the lack of sensitivity and selectivity of these methods. Therefore, an efficient separation procedure is usually required prior to the determination of noble metals for sensitive, accurate, and interference-free determination of noble metals.

Several analytical methods have been utilized for separation of analyte of interest, including liquid/liquid extraction, ion exchange, coprecipitation, cloud-point extraction, and solid-phase extraction (SPE) [11,12]. SPE is considered to be one of the most powerful techniques because it minimizes solvent usage and exposure, disposal costs, and extraction time for sample preparation. Several adsorbents have appeared because of the popularity of SPE for selective extraction of analytes such polymers, silica, carbon nanotube, etc. [7,8].

Nanoscience and technology have attracted significant attention due to its potential application in various fields and especially in metal ion adsorption [13,14]. ZnO, a versatile material, emerges as a challenging prospect in the field of nanotechnology. Nanosized ZnO has been widely used as a catalyst [14], gas sensor [15,16], active filler for rubber and plastic, ultraviolet (UV) absorber in cosmetics, and antivirus agent in coating [17,18] and has more potential application in building functional electronic devices with special architecture and distinctive optoelectronic properties.

In this investigation, we synthesized ZnO nanosheets by stirring method and characterized by X-ray diffraction patterns (XRD), field emission scanning electron microscopy (FESEM), Fourier transform infrared spectroscopy (FT-IR), X-ray photoelectron spectroscopy (XPS), and energy dispersive spectroscopy (EDS). ZnO nanosheets were applied to investigate their utility and the analytical efficiency as adsorbent on the selectivity and adsorption capacity of Cd(II). The selectivity of ZnO nanosheets toward eight metal ions, including Cd(II), Cu(II), Hg(II), La(III), Mn(II), Pb(II), Pd(II), and Y(III), was investigated in order to study the effectiveness of ZnO nanosheets on the adsorption of selected metal ions. Based on the selectivity study, the ZnO nanosheets attained the highest selectivity toward Cd(II). Static uptake capacity of ZnO nanosheets for Cd(II) was found to be 97.36 mg g^−1^. Adsorption isotherm data of Cd(II) with ZnO nanosheets were well fit with the Langmuir adsorption isotherm, strongly confirming that the adsorption process was mainly monolayer on homogeneous adsorbent surfaces.

## Methods

### Chemicals and reagents

Zinc nitrate, sodium hydroxide, mercuric nitrate, lanthanum nitrate, palladium nitrate, and yttrium nitrate were purchased from Sigma-Aldrich (Milwaukee, WI, USA). Stock standard solutions of 1,000 mgL^−1^ Cd(II), Cu(II), Mn(II), and Pb(II) were also obtained from Sigma-Aldrich. All reagents used were of high purity and of spectral purity grade, and doubly distilled deionized water was used throughout.

### Preparation of ZnO nanosheets

ZnO nanosheets were synthesized by thermal stirring method in which 0.1 M of zinc nitrate aqueous solution was titrated with 0.1 M solution of NaOH till pH reached above 10 and stirred at 70°C for overnight. White product was washed and dried. The dried product was calcined at 450°C for 4 h.

### Possible growth mechanism of ZnO nanosheets

The formations of ZnO might take place by following probable chemical reactions:

$$\begin{array}{r} {{\text{NaOH}\to \text{Na}}^{+}+}^{-}\mathrm{OH}\ldots ..\ldots ..\ldots ..\ldots \ldots ..\ldots \left(1\right)\\ {\mathrm{Zn}\left(NO_{3}\right)}_{2}\to {\text{Zn}}^{2+}+{{2\mathrm{NO}}_{3}}^{-}..\ldots ..\ldots ..\ldots ..\ldots ..\left(2\right)\\ {\text{Zn}}^{2+}+{2\text{OH}}^{-}\to {\mathrm{Zn}(\text{OH})}_{2}..\ldots ..\ldots ..\ldots ..\ldots ..\ldots \left(3\right)\\ {\text{Zn}(\mathrm{OH})}_{2}\to \mathrm{ZnO}..\ldots ..\ldots ..\ldots ..\ldots \ldots ..\ldots ..\left(4\right) \end{array}$$

**Figure 1:**
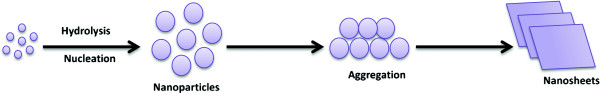
Schematic representation of ZnO nanosheets growth mechanism.

Initially, Zn(NO_3_)_2_ and NaOH undergo hydrolysis in water and produce Zn^2+^ and OH^−^ which later produce Zn(OH)_2_. The heating causes the dehydration of Zn(OH)_2_ (orthorhombic structure) to ZnO (monoclinic structure). During the growth process (Figure 1), first ZnO nucleus growth takes place which then aggregates and produces ZnO nanoparticles by Ostwald ripening. Nanoparticles crystallize and aggregate with each other through Van der Waals forces and hydrogen bonding and give ZnO nanosheets.

### Characterization

The morphology of the synthesized product was studied at 15 kV using a JEOL Scanning Electron Microscope (JSM-7600 F, Akishima-shi, Japan). XRD was taken with a computer-controlled RINT 2000, Rigaku diffractometer (Shibuya-ku, Japan) using the Ni-filtered Cu-Kα radiation (*λ* = 0.15405 nm). FT-IR spectrum was recorded in the range of 400 to 4,000 cm^−1^ on PerkinElmer (spectrum 100, Waltham, MA, USA) FT-IR spectrometer. XPS spectrum was recorded in the range of 0 to 1,350 eV by using Thermo Scientific K-Alpha KA1066 spectrometer (Schwerte, Germany).

### Samples preparation and procedure for metal uptake study

Stock solutions of Cd(II), Cu(II), Hg(II), La(III), Mn(II), Pb(II), Pd(II), and Y(III) were prepared in 18.2 MΩ·cm distilled deionized water and stored in the dark at 4°C. For studying the selectivity of ZnO nanosheets toward metal ions, standard solutions of 2 mg L^−1^ of each metal ion were prepared and adjusted to pH value of 5.0 with a buffered aqueous solution (0.1 mol L^−1^ CH_3_COOH/CH_3_COONa). Standard solutions were adjusted at pH value of 5.0 in order to avoid the formation of suspended gelatinous lanthanides hydroxides with buffer solutions at pH values beyond 5.0. Each standard solution was individually mixed with 25 mg of the ZnO nanosheets. For investigation of the Cd(II) adsorption capacity, standard solutions of 0, 5, 10, 15, 20, 25, 30, 50, 75, 125, and 150 mg L^−1^ were prepared as above, adjusted to pH value of 5.0 and individually mixed with 25 mg ZnO nanosheets. All mixtures were mechanically shaken for 1 h at room temperature.

Inductively coupled plasma-optical emission spectrometry (ICP-OES) measurements were acquired by use of a Perkin Elmer ICP-OES model Optima 4100 DV (Waltham, MA, USA). The ICP-OES instrument was optimized daily before measurement and operated as recommended by the manufacturers. The ICP-OES spectrometer was used with following parameters: FR power, 1,300 kW; frequency, 27.12 MHz; demountable quartz torch, Ar/Ar/Ar; plasma gas (Ar) flow, 15.0 L min^−1^; auxiliary gas (Ar) flow, 0.2 L min^−1^; nebulizer gas (Ar) flow, 0.8 L min^−1^; nebulizer pressure, 2.4 bars; glass spray chamber according to Scott (Ryton), sample pump flow rate, 1.5 mL min^−1^; integration time, 3 s; replicates, 3; wavelength range of monochromator, 165 to 460 nm. Selected metal ions were measured at wavelengths of 228.80 nm for Cd(II), 327.39 nm for Cu(II), 194.17 nm for Hg(II), 348.90 nm for La(III), 275.61 nm for Mn(II), 220.35 nm for Pb(II), 340.46 nm for Pd(II), and 361.10 nm for Y(III).

## Results and discussion

### Structural characterization

**Figure 2:**
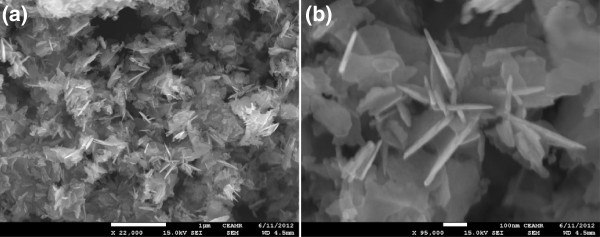
Typical (a) low-magnification and (b) high-resolution FESEM images of ZnO nanosheets.

FESEM was used for the general structural characterization of the calcined products and demonstrated in Figure 2. It is clear from the images that the synthesized product is grown in high density. The calcined product possess sheet like structure and average thickness of the grown nanosheets is approximately 10 nm.

**Figure 3:**
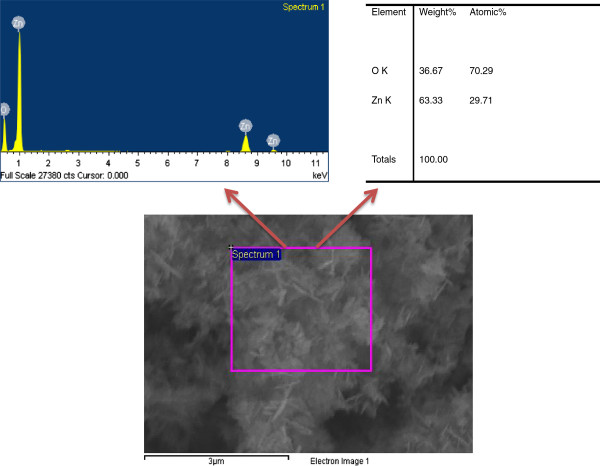
Typical EDS spectrum of ZnO nanosheets.

The chemical composition of the synthesized nanosheets was studied by energy dispersive spectroscopy (EDS), and the results were depicted in Figure 3. The EDS did not show any element except zinc and oxygen which suggest that the synthesized nanosheets are pure ZnO.

**Figure 4:**
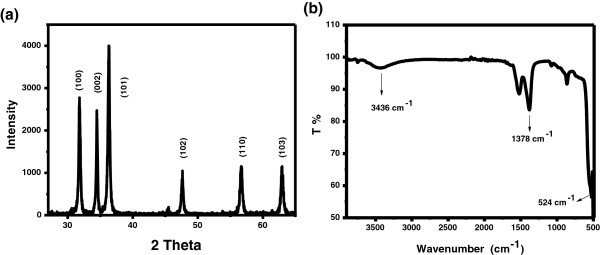
Typical (a) XRD pattern and (b) FT-IR spectrum of ZnO nanosheets.

To check the crystallinity of the synthesized ZnO nanosheets, X-ray diffraction technique was used, and results are shown in Figure 4a. A series of characteristic peaks were obtained which are responsible for wurtzite hexagonal ZnO. X-ray diffraction confirms that the obtained nanomaterial is pure ZnO with wurtzite hexagonal phase [19].

Figure 4b shows the typical FT-IR spectra of the ZnO nanomaterial measured in the range of 420 to 4,000 cm^−1^. The appearance of a sharp band at 495.18 cm^−1^ in the FT-IR spectrum is indication of ZnO nanosheets which is due to Zn-O stretching vibration [19]. The absorption peaks at 3,477 and 1,612 cm^−1^ are caused by the O-H stretching of the absorbed water molecules from the environment [20].

**Figure 5:**
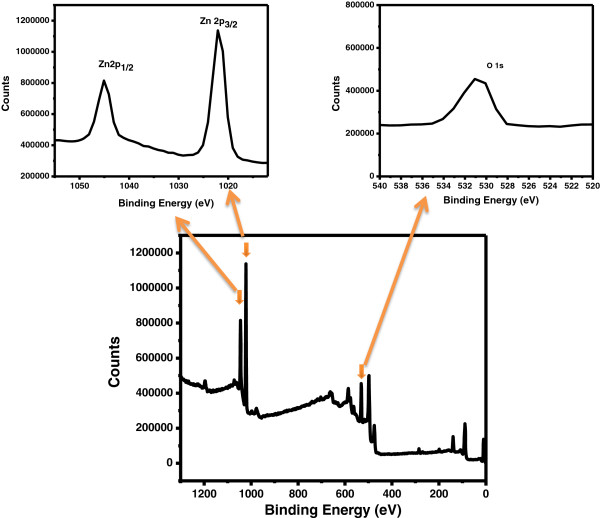
Typical XPS spectrum of ZnO nanosheets.

XPS was analyzed for synthesized nanosheets and described in Figure 5. XPS peaks for calcined nanosheets observed at 531.1 for O 1 s, 1,022.0 eV for Zn 2p_3/2_, and 1,045.0 eV for Zn 2p_1/2_ which are comparable to the literature values [21] which suggest pure ZnO nanosheets.

### Metal uptake

#### Selectivity study of ZnO nanosheets

Selectivity of the newly synthesized ZnO nanosheets toward different metal ions was investigated based on the basis of calculated distribution coefficient of ZnO nanosheets. The distribution coefficient (*K*_d_) can be obtained from the following equation [22]:

(1) $$K_{d}=\left(C_{o}–C_{e}/C_{e}\right)\times \left(V/m\right),$$

where *C*_o_ and *C*_e_ refer to the initial and final concentrations before and after filtration with ZnO nanosheets, respectively, *V* is the volume (mL), and *m* is the weight of ZnO nanosheets (g). Distribution coefficient values of all metal ions investigated in this study are summarized in Table 1. It can be clearly observed from Table 1 that the greatest distribution coefficient value was obtained for Cd(II) with ZnO nanosheets in comparison to other metal ions. As can be depicted from Table 1, the amount of Cd(II) was almost all extracted using ZnO nanosheets. Thus, selectivity study results indicated that the newly synthesized ZnO nanosheets were most selective toward Cd(II) among all metal ions. The incorporated donor atom of oxygen, presented in ZnO nanosheets, strongly attained the selective adsorption of ZnO nanosheets toward Cd(II). Based on the above results, the mechanism of adsorption may be electrostatic attraction or chelating mechanism between ZnO nanosheets and Cd(II).

**Table 1:** **Selectivity study of ZnO nanosheets adsorption toward different metal ions at pH 5.0 and 25°C (*N*= 5)**

**Metal ion**	***q***_**e**_**(mg g**^**−1**^**)**	***K***_**d**_**(mL g**^**−1**^**)**
Cd(II)	1.98	89,909.09
Mn(II)	1.53	3,237.29
Cu(II)	1.41	2,412.97
Y(III)	1.33	1,985.07
Pb(II)	1.25	1,666.67
La(III)	1.08	1,166.85
Hg(II)	0.73	568.63
Pd(II)	0.35	209.19

#### Static adsorption capacity

**Figure 6:**
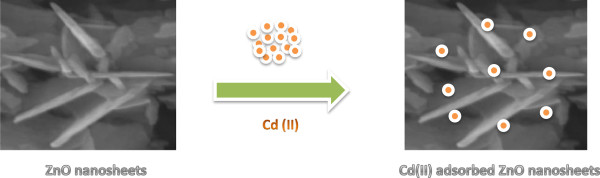
Schematic view of Cd(II) adsorption process on ZnO nanosheets.

**Figure 7:**
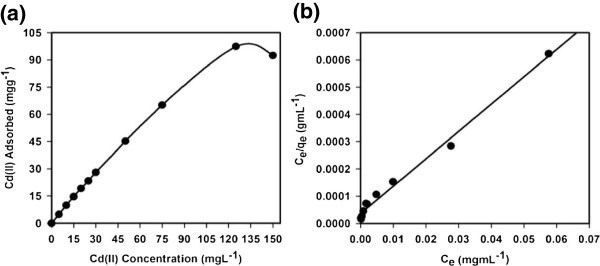
**Adsorption profile of Cd(II) (a) and Langmuir adsorption isotherm model of Cd(II) adsorption (b).** On 25 mg of ZnO nanosheets at pH 5.0 and 25°C. Adsorption experiments were obtained at different concentrations (0 to 150 mg L^−1^) under static conditions.

For determination of the static uptake capacity of Cd(II) on ZnO nanosheet adsorbent, 25 mL Cd(II) sample solutions with different concentrations (0 to 150 mg L^−1^) were adjusted to pH 5.0 and individually mixed with 25 mg ZnO nanosheets (Figure 6). These mixtures were mechanically shaken for 1 h at room temperature. Static adsorption capacity was obtained using Equation 2 as follows:

(2) $$q_{e}=\frac{\left(C_{ο}-C_{e}\right)\;V}{m},$$

where *q*_e_ represents the adsorbed metal ion by the adsorbent (mg g^−1^), *C*_o_ and *C*_e_ are the initial and equilibrium concentrations of metal ion in solution (mg L^−1^), respectively, *V* is the volume (L), and *m* is the weight of adsorbent (g). Figure 7a displays the metal uptake capacity of ZnO nanosheets for Cd(II) obtained from the experiment of adsorption isotherm. Adsorption capacity of ZnO nanosheets for Cd(II) was determined to be 97.36 mg g^−1^. Reported adsorption capacity in this study was found to be comparable with those previously reported for Cd(II) (4.92 [23], 9.39 [24], 84.30 [25], 57.90 [26], 95.20 [27], 123.65 mg g^−1^[28]) in other studies. In comparison with the adsorption capacity of ZnO nanosheets toward Cd(II), uptake capacities of other nanostructures for Cd(II) were also previously reported. For example, the adsorption capacity of Cd(II) on MnO_2_ functionalized multi-walled carbon nanotubes was determined to be 41.60 mg g^−1^ by Luo et al. [29]. In addition, adsorption capacities of nano B_2_O_3_/TiO_2_ composite material and nanocrystallite hydroxyapatite for Cd(II) were previously evaluated and reported to be 49.00 [30] and 142.86 mg g^−1^[31]. As discussed above, the adsorption capacity of nanostructures for Cd(II) may vary. However, ZnO nanosheets possess the most important property in its high efficiency and selectivity for Cd(II). Thus, the high selectivity of ZnO nanosheets enables the method for accurate and precise determination of Cd(II) in complex matrices.

#### Adsorption isotherm models

Experimental equilibrium adsorption data were analyzed using different models in order to develop an equation that accurately represents the results. Langmuir equation is based on an assumption of a monolayer adsorption onto a completely homogeneous surface with a finite number of identical sites and a negligible interaction between the adsorbed molecules. The Langmuir adsorption isotherm model is governed by the following relation [7]:

(3) $$C_{e}/q_{e}=\left(C_{e}/Q_{o}\right)+1/Q_{o}b,$$

where *C*_e_ corresponds to the equilibrium concentrations of Cd(II) ion in solution (mg mL^−1^) and *q*_*e*_ is the adsorbed metal ion by the adsorbate (mg g^−1^). The symbols *Q*_o_ and *b* refer to Langmuir constants related to adsorption capacity (mg g^−1^) and energy of adsorption (L mg^−1^), respectively. These constants can be determined from a linear plot of *C*_e_/*q*_e_ against *C*_e_ with a slope and intercept equal to 1/*Q*_o_ and 1/*Q*_o_*b*, respectively. Moreover, the essential characteristics of Langmuir adsorption isotherm can be represented in terms of a dimensionless constant separation factor or equilibrium parameter, *R*_L_, which is defined as *R*_L_ = 1/(1 + *bC*_o_), where *b* is the Langmuir constant (indicates the nature of adsorption and the shape of the isotherm); *C*_o_ the initial concentration of the analyte. The *R*_L_ value indicates the type of the isotherm, and *R*_L_ values between 0 and 1 represent a favorable adsorption [8].

The experimental isotherm data were best fit with the Langmuir equation (Figure 7b) based on the least square fit, confirming the validity of Langmuir adsorption isotherm model for the adsorption process. Consequently, adsorption isotherm data suggested that the adsorption process was mainly monolayer on a homogeneous adsorbent surface. Langmuir constants *Q*_o_ and *b* are found to be 99.60 mg g^−1^ and 0.28 L mg^−1^, respectively. The correlation coefficient obtained from the Langmuir model is found to be *R*^2^ = 0.989 for adsorption of Cd(II) on ZnO nanosheets. Moreover, the Cd(II) adsorption capacity (99.60 mg g^−1^) calculated from Langmuir equation was consistent with that (97.36 mg g^−1^) of the experimental isotherm study. The *R*_L_ value of Cd(II) adsorption on the ZnO nanosheets is 0.03, supporting a highly favorable adsorption process based on the Langmuir classical adsorption isotherm model.

## Conclusions

ZnO nanosheets were synthesized by low-temperature eco-friendly method and evaluated their efficiency for selective adsorption and determination of Cd(II) in aqueous solution. Reasonable static adsorption capacities of 97.36 mg g^−1^ for ZnO nanosheet adsorbent were achieved for Cd(II) in aqueous solution. Adsorption isotherm data of Cd(II) were well fit with the Langmuir classical adsorption isotherm model. Thus, the method may play an important role for using it as an effective approach for a selective adsorption and determination of Cd(II) in complex matrices for a range of several applications.

## Competing interests

The authors declare that they have no competing interests.

## Authors’ contribution

SBK and MMR synthesized the ZnO nanosheets, performed structural analyses of the samples, analyzed the experimental results, and contributed to the manuscript preparation. AMA and KAA coordinated the study, analyzed the data, and contributed to the manuscript preparation. HMM carried out the metal ion adsorption study and analyzed the data and contributed to the manuscript preparation. All authors read and approved the final manuscript.
